# Pollicisation: our techniques

**DOI:** 10.1186/1753-6561-9-S3-A7

**Published:** 2015-05-19

**Authors:** Bhaskaranand Kumar

**Affiliations:** 1 Division of Hand and Microsurgery, Kasturba Medical College, Manipal, 576104 India

## 

Pollicization remains one of the most useful surgeries in the field of hand surgery as it involves creation of a new thumb which contributes to 40 percent of hand function. It involves transfer of one of the fingers to create a new functioning thumb when it is absent. The surgery demands an intelligent use of skin flaps, osteotomy and muscle balancing to create an almost normal looking aesthetic thumb.

The principles advocated by Buck-Gramcko remain as standards for us to adhere. This involves: 1) A carefully planned skin incision, 2) Neurovascular Dissection, 3) Skeletal readjustment to achieve length, orientation and alignment and 4) Optimum muscle balance to get the strength and stability for the new thumb.

We present a review based on experience of more than 200 pollicizations, briefly discussing indications, refinements in the critical steps of the technique, long term results and complications. Pollicization continues to be one of the most useful surgeries in improving the function of the hand and has stood the test of time. Its long term results clearly show that patients across all age groups with different etiologies use the pollicized thumb activity.

